# A Bioactive Chemical Markers Based Strategy for Quality Assessment of Botanical Drugs: Xuesaitong Injection as a Case Study

**DOI:** 10.1038/s41598-017-02305-y

**Published:** 2017-05-25

**Authors:** Zhenzhong Yang, Qing Shao, Zhiwei Ge, Ni Ai, Xiaoping Zhao, Xiaohui Fan

**Affiliations:** 1 Pharmaceutical Informatics Institute, College of Pharmaceutical Sciences, Zhejiang University, Hangzhou, 310058 China; 2 College of Preclinical Medicine, Zhejiang Chinese Medical University, Hangzhou, 310053 China

## Abstract

Current chemical markers based quality assessment methods largely fail to reflect intrinsic chemical complexity and multiple mechanisms of action of botanical drugs (BD). The development of novel quality markers is greatly needed. Here we propose bioactive chemical markers (BCM), defined as a group of chemo-markers that exhibit similar pharmacological activities comparable to the whole BD, which can therefore be used to effectively assess the quality of BD. As a proof-of-concept, a BCM-based strategy was developed and applied to Xuesaitong Injection (XST) for assessing the efficacy and consistency of different batches. Firstly, systemic characterization of chemical profile of XST revealed a total number of 97 compounds. Secondly, notoginsenoside R_1_, ginsenoside Rg_1_, Re, Rb_1_ and Rd were identified as BCM of XST on treating cardiovascular and cerebrovascular diseases according to Adjusted Efficacy Score following an *in vivo* validation. Analytical method for quantification of BCM was then developed to ensure the efficacy of XST. Finally, chemical fingerprinting was developed and used to evaluate the batch-to-batch consistency. Our present case study on XST demonstrates that BCM-based strategy offers a rational approach for quality assessment of BD and provides a workflow for chemistry, manufacturing, and controls (CMC) study of BD required by regulatory authority.

## Introduction

The trend of returning back to nature spreads throughout the world, which has stimulated great enthusiasm in the development of botanical drugs for the prevention and treatment of complex diseases^1, 2^. Different from chemical drugs with single active pharmaceutical ingredient (API), botanical drugs usually consist of complex mixture of phytochemical constituents, which raises a serious issue in their quality assurance^3^. Benefit from the rapid development of the analysis methods and techniques, nowadays it is not a difficulty to characterize and quantify the chemical constituents in botanical drugs. For example, hundreds of constituents have been identified^4, 5^, as well as more than a dozen constituents have been quantified^6, 7^ in *Panax notoginseng* (Burk.) F. H. Chen. Regulatory authority has also put forward further requirements for illumination of the chemical composition in botanical drugs, e.g., China Food and Drug Administration (CFDA) has issued *Basic Technical Requirements for TCM Injections and Natural Medicine Injections* ^8^. In this *Requirements*, criteria for injections made up of multiple compounds are as follows: Accumulative content of identified constituents should be more than 60% of total content, accumulative content of constituents with accurate quantification should be more than 80%, and accumulative content of identified constituents in the reference fingerprint should be more than 90%.

However, it is not applicable to determine all the constituents in botanical drugs, due to the economic factor. For botanical drug substance and drug product, current quality control tests mainly involves identification and quantification of individual chemical markers to ensure the quality of these phyto-therapeutic agents. The critical issue is to clarify which substances to control and how to control them, which belongs to an essential portion of the chemistry, manufacturing, and controls (CMC) information required for new drug application in many countries including USA and China^9, 10^. In many cases, these chemistry-oriented methods select constituents with relatively high contents or those that are easy to be measured as chemical markers, however correlations between their quantities in the drugs and therapeutic activities are not well established^11^. Subsequently, biological assays are developed and adopted to evaluate the quality of some botanical drugs^12, 13^. Another question is raised up for this group of methods whether a single biological assay can properly reflect integrative therapeutic effects of botanical drugs for treating complex diseases. Thus, novel and effective methods for quality assessment of botanical drugs are greatly needed.

Botanical drugs usually exhibit therapeutic effects via a group of active constituents. Bioactive chemical markers (BCM) are defined here as a group of chemo-markers with overall therapeutic activity that is substantially comparable to that of the botanical drug. Ensuring the efficacy of the botanical drug can be achieved by controlling the contents of these BCM, which suggests a set of promising quality assessment/control markers representing pharmacological characteristics of botanical drugs. Through the integration of biology and chemistry perspective together, a new venue on quality assessment of botanical drugs may be open to manufacturing industry and regulatory agencies. Numerous studies have been conducted to investigate active components of botanical drugs^14–19^, however there is still lack of practical and effective methods for identifying their BCM, especially for those treating multi-factor complex diseases, such as cardiovascular and cerebrovascular diseases (CCVD).

In addition to BCM, many minor constituents always present in the botanical drugs. It is notable that relative contents of these minor constituents can vary from batch to batch, which might not exert obvious influence on overall therapeutic efficacy. However potential risk to the health of the patient still needs to be considered. Thus, comprehensive characterization of drug substance is important. To warrant the batch-to-batch consistency of botanical product, it is also critical to limit the fluctuation of minor constituents within a certain range. As a widely accepted technique to evaluate batch-to-batch consistency of botanical drugs^9, 20, 21^, chemical fingerprinting is an important component of quality assessment system in which quantity alterations of minor constituents are taken into account^22, 23^.

**Figure 1:**
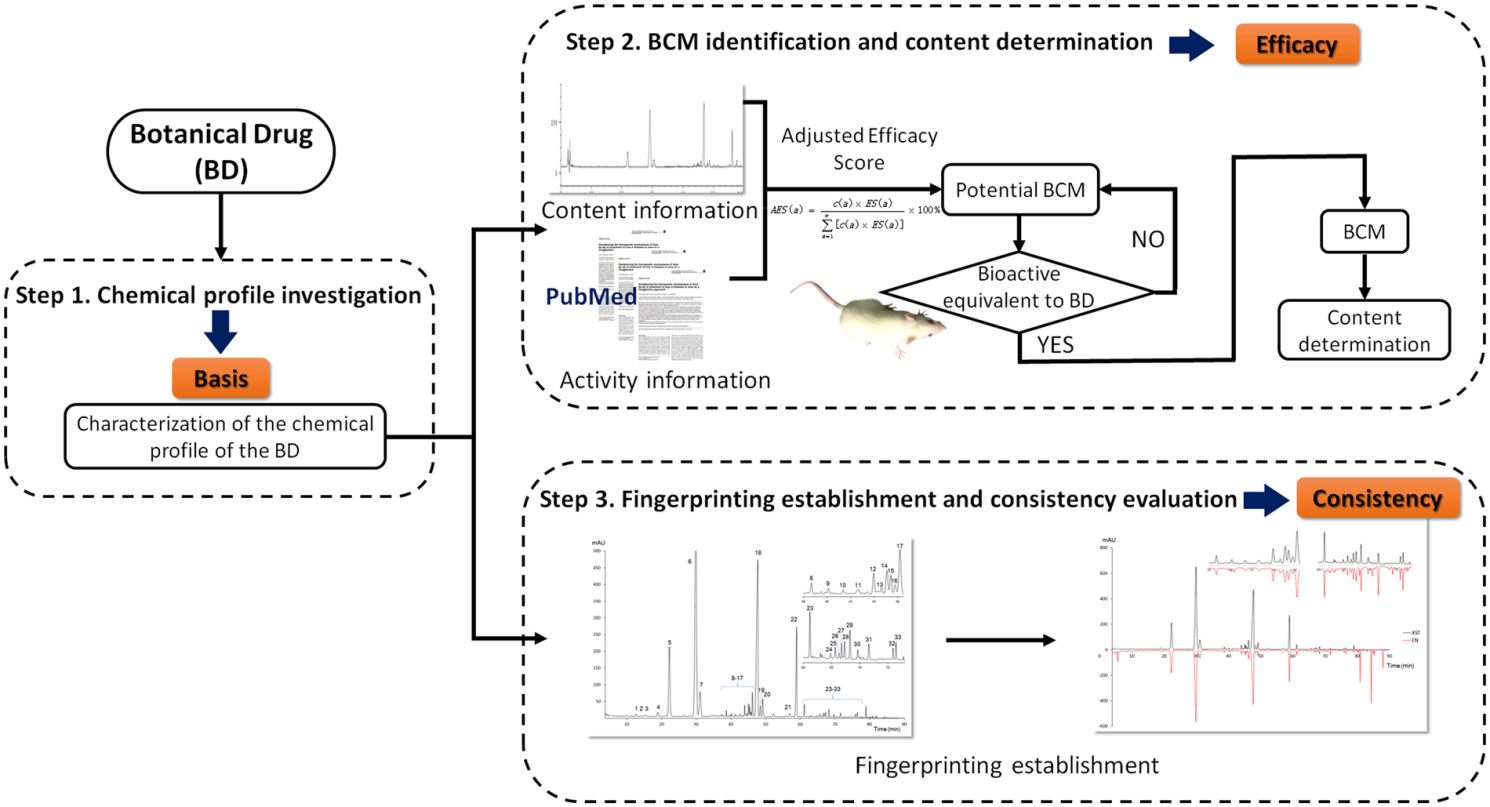
The flowchart of the bioactive chemical markers based quality assessment strategy for botanical drugs.

Xuesaitong Injection (XST) is one of the best-selling botanical drugs in the Chinese pharmaceutical market, which is made of the total saponins from *P. notoginseng*. XST has been extensively used in the treatment of CCVD in China^24, 25^. Although a number of studies have reported chemical components in *P. notoginseng* ^4, 26^, the chemical profile of XST remains to be investigated comprehensively, especially regarding to these minor and trace constituents. What is the BCM of XST and which constituents should be measured in the quality assessment of XST, have not been elaborated yet. A novel and effective strategy was proposed in this study for quality assessment of botanical drugs, as shown in Fig. 1. The constituents in the botanical drug should first be investigated thoroughly, which served as the basis for subsequent two steps. Then, the BCM of the botanical drug are uncovered by Adjusted Efficacy Score based method, and verified by *in vivo* experiments. Quantitative determination of the contents of BCM provides important data for quality assessment based on the efficacy of the botanical drug. Finally, fingerprinting should be established for assessing the batch-to-batch consistency of the final product. As a proof-of-concept, this strategy was applied to quality assessment of XST as a case study.

## Results and Discussion

### Characterization of the constituents in XST

**Figure 2:**
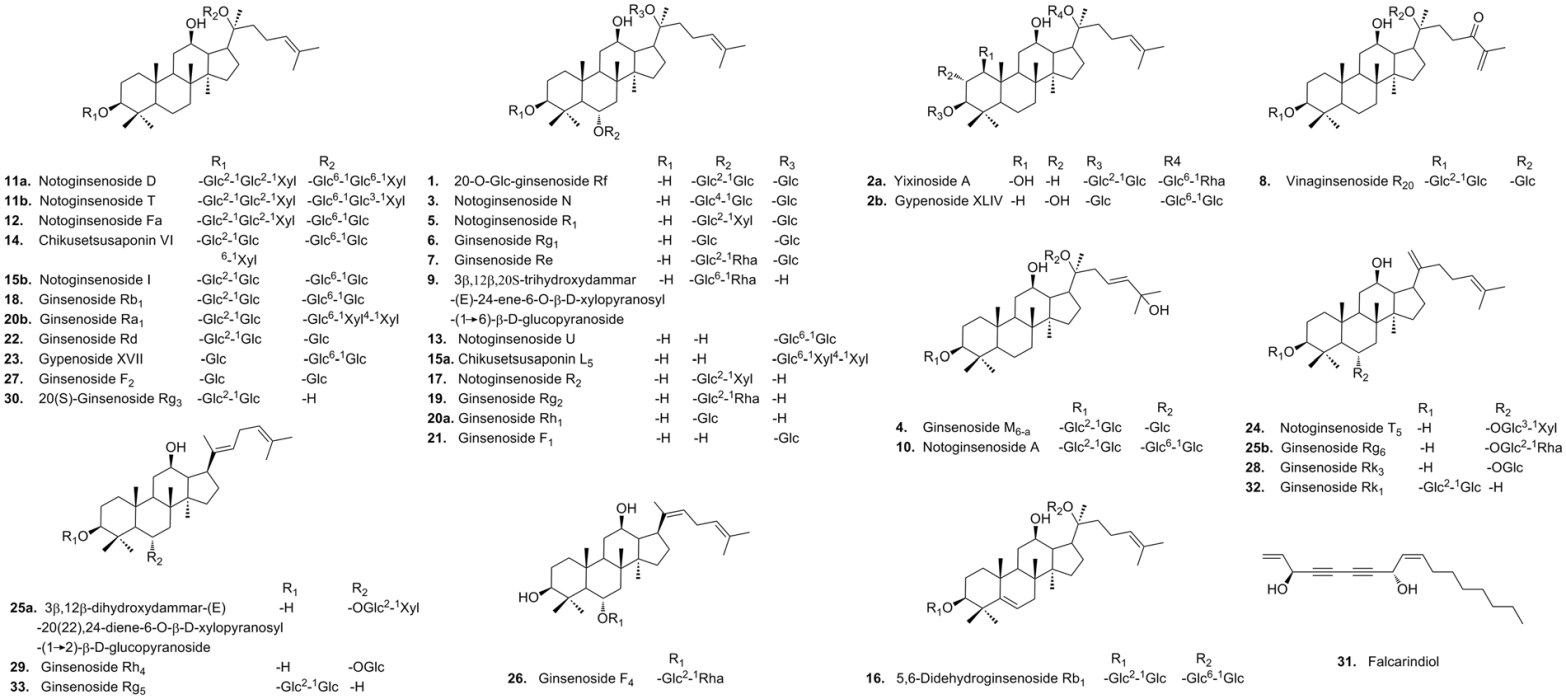
Typical chemical structures of constituents in XST.

XST is consisted of the total saponins from *P. notoginseng*. Numerous analytical methods have been applied for qualitative or quantitative analysis of its major constituents, i.e., NG-R_1_, G-Rg_1_, G-Re and G-Rb_1_, etc. However, comprehensive investigation of chemical profile of XST, especially these minor and trace constituents, has rarely been performed. Considering their potential importance for the quality control of XST, the constituents of XST were investigated thoroughly here. XST lyophilized powder was first dissected to 10 fractions by preparative HPLC to decrease the complexity and enrich the minor and trace constituents. These 10 fractions were then analyzed by HPLC-MS^n^ for the constituent characterization. Fragmentation rules, summarized from the analysis of the available reference standards and the literatures, were used to characterize these constituents. A total of 97 constituents were characterized or tentatively identified by comparing the retention times, and MS^n^ data of the constituents with available reference standards or published information (summarized in Table S1). The typical chemical structures of constituents in XST were shown in Fig. 2.

### Potential BCM identified by Adjusted Efficacy Score

**Figure 3:**
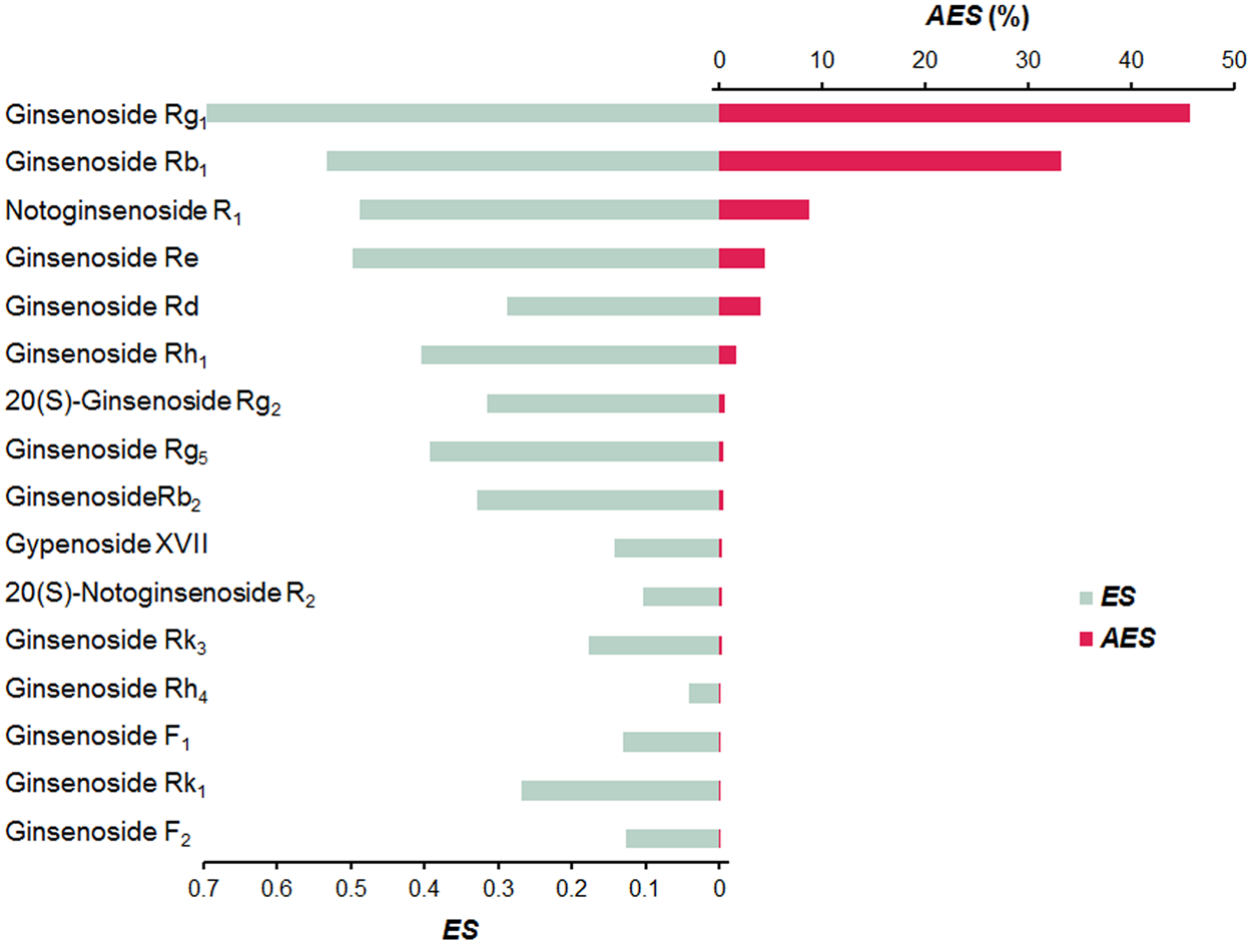
The ES and AES of the constituents in XST.

For the majority of botanical drugs, active constituents responsible for therapeutic activity were still unknown. As a common practice in quality assessment of these drugs, constituents with relatively high content are usually employed as chemical markers, regardless of the relationship with the botanical drugs’ therapeutic effects. Botanical drugs, especially these for the multi-factor complex disease, such as CCVD, are characterized to exert therapeutic activities via multi-components and complex mechanisms of action. In order to develop efficacy-oriented quality assessment/control markers for botanical drugs, the concept of BCM has been proposed. However, lack of appropriate methods for the BCM identification limits its real-world application. Because both the activity of the constituent and its content would influence the role that this constituent plays in the botanical drug. Herein, a BCM identification method based on Adjusted Efficacy Score (AES) was developed for uncovering the BCM in botanical drugs. In our previous study, therapeutic action of XST against myocardial infarction was through “the multi-compound, multi-target and multi-pathway” manner^27^, and major active constituents of XST against cardiovascular diseases were identified based on the contents and predicted protein targets of constituents^28^. In addition to acute myocardial infarction, XST is also clinically used in the treatment of cerebral infarction, thrombosis, and other CCVD. In order to identify BCM representing clinical efficacy of XST, a broad view on diverse activities in CCVD treatment should be considered into the BCM determination. In this study, the anti-CCVD effects of XST were comprehensively summarized as seven main aspects (described in the Experimental section), including myocardial protection, anticoagulation, antihypertension, anti-inflammation, etc^29^. Due to its excellent therapeutic performance of XST in the clinic, associated effects of many constituents have been well studies on their protective mechanisms against CCVD. Based on manual data mining on available literatures, the anti-CCVD effects of these constituents were investigated and summarized here (more details listed in Table S2). An index named Efficacy Score (ES) was first developed to indicate the integrated anti-CCVD effect of these constituents. Taking the content of the constituent in botanical drugs into consideration, AES was then employed to represent the importance of each constituent in botanical drugs for overall therapeutic efficacy. In most literatures, 20(S)-G-Rh_1_ and 20(R)-G-Rh_1_ were not definitely differentiated, and their activity and content data were combined as that of G-Rh_1_. Information about the contents of saponins in XST previously determined in our group through HPLC^28^ was adopted in this study. As shown in Fig. 3, the AESs of G-Rg_1_, G-Rb_1_, NG-R_1_, G-Re and G-Rd were 45.7, 33.1, 8.7, 4.5 and 4.0%, respectively. The accumulated AES of these five constituents already reached 96.0%. Therefore, these five saponins were selected as potential BCM which was subject to comparison study *in vivo*.

### Experimental verification of BCM *in vivo*

**Figure 4:**
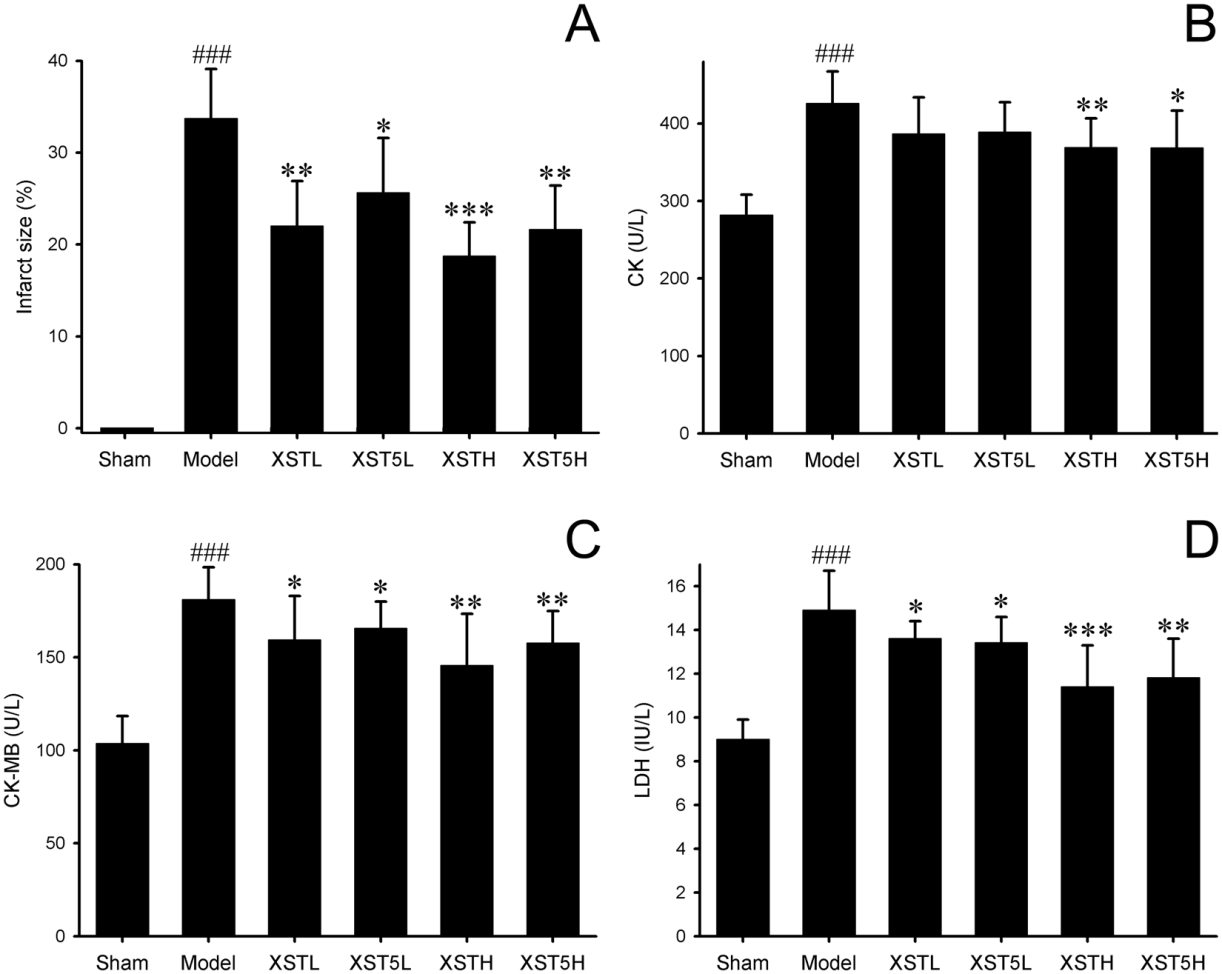
The effect of XST and XST5 in myocardial infarction. (**A**) The effect on infarct size (n = 6). The effect on CK (**B**), CK-MB (**C**) and LDH (**D**) activity (n = 10). All values are means ± SD. ^###^ *p* < 0.001 vs. Sham group, *p < 0.05 vs. Model group, **p < 0.01 vs. Model group, ***p < 0.001 vs. Model group.

Myocardial ischemia is one of main clinical indications of XST^24, 27^. To verify potential BCM of XST identified by AES, these five saponins were combined manually based on their contents in the injection and *in vivo* activity against myocardial ischemia was evaluated for this BCM mixture then compared with XST by the classic left coronary artery ligation MI model^30^. Compared with Model group, XST treatment could dose dependently decrease the infarct size, as shown in Fig. 4A. Meanwhile, anti-myocardial ischemia effect of five main saponins in XST (XST5), i.e., NG-R_1_, G-Rg_1_, G-Re, G-Rb_1_ and G-Rd, was also evaluated *in vivo*. Both XST5H and XST5L displayed significant cardio-protective activity in MI rats as shown by decreased infarction size. There are no significant difference in anti-MI effect observed between XST5 and XST in this experiment. With the presence of MI, the activities of myocardial marker enzymes in serum including CK, CK-MB and LDH also increased, indicating the presence of myocardial damage. As shown in Fig. 4B,C and D, both high dose XST and XST5 treatment could significantly alleviate the injury caused by the ligation and their effect was comparable as shown by biochemistry results. From the *in vivo* results mentioned above, XST5 performed a comparable anti-myocardial ischemia effect with the whole XST. Therefore, XST5 were recognized as the BCM of XST, which can be regarded as quality assessment/control markers in further quality assessment methods to ensure the efficacy of XST. In other words, the BCM of XST would be determined to ensure the clinical efficacy of XST instead of the complicated *in vivo* experiments.

### Method validation and quantitative determination of BCM in multiple batches of XST samples

The quantification method of BCM in XST was validated in terms of linearity, precision, repeatability, stability and accuracy. Series of standard solutions comprising the five saponins were prepared in 50% methanol and were used to determine the linearity of the analytes. The results of calibration were summarized in Table 1. Within test ranges, good correlations were found between the peak area (y) and concentration of each analytes (x) (r > 0.9996). Precisions were determined by analyzing the six replicate samples. The results indicated that the relative standard deviation (RSD) values of the retention time and peak area were less than 0.5% and 1.2%, respectively. For repeatability, six independently prepared solutions of the same sample (S1) were analyzed. RSD values of the contents of the five saponins were less than 1.6%. For the stability test, a sample was prepared and analyzed 0, 3, 6, 9, 12, 18 and 24 hours after preparation, respectively. The RSD values of the peak area of five saponins were less than 1.9%. This suggested that it was feasible to analyze samples within 24 h. The accuracy was validated by the recovery test through standard addition method. The samples were spiked with a known amount (high, mediate and low levels) of standard solution, and then analyzed by the proposed method. Each set of samples was analyzed for three times. The developed method was reproducible with average recovery within the range of 98.59–101.4% with RSD ≤ 3.10%.

**Table 1:** Linear regression data of BCM in XST.

Compound	Calibration curve	Linearity range (mg/mL)	Correlation coefficient (r)
NG-R_1_	y = 1481.0x − 5.3	0.1245–1.992	0.9999
G-Rg_1_	y = 1733.2x − 3.9	0.4424–7.078	1.0000
G-Re	y = 1508.2x − 9.2	0.1311–2.097	1.0000
G-Rb_1_	y = 1273.7x − 0.7	0.4388–7.021	1.0000
G-Rd	y = 1551.7x + 2.2	0.1246–1.994	0.9997

The quantitative method developed here was subsequently applied to the simultaneous determination of the BCM in XST. Eight batches of XST were employed, and each sample was determined in triplicate. The quantitative results were summarized in Table S3. Results indicated that the BCM (including NG-R_1_, G-Rg_1_, G-Re, G-Rb_1_ and G-Rd) accounted for more than 85% of the total contents. Eight batches of samples displayed the acceptable variation with RSD <4.7% for both of contents of each constituents and accumulative contents of five BCM, which suggests efficacies of these batches of XST can be ensured by controlling these BCM.

### HPLC condition optimization for chemical fingerprinting analysis of XST

To achieve the optimal separation and obtain more chemical information, the influence of different columns, mobile phases, column temperature, detection wavelength, injection volume, flow rate and gradient program in chemical fingerprinting of XST were investigated. Three kinds of reversed-phase columns, including Lichrospher C_18_ (4.6 mm × 250 mm, 5 µm), Agilent Zorbax Eclipse XDB-C_18_ (4.6 mm × 250 mm, 5 µm), Agilent Zorbax SB-C_18_ (4.6 mm × 250 mm, 5 µm), were compared. Agilent Zorbax SB-C_18_ column exhibited better separation, and was chosen for further research. The wavelengths of 203, 210, 254, 280 and 320 nm were selected for comparing the peak numbers detected. Because the peaks were mainly derived from saponins, the chromatogram monitored at 203 nm revealed more peaks than other wavelengths. Therefore, 203 nm was chosen as the detection wavelength. Different mobile phases on chromatographic separation were investigated. Aqueous with 0.01% acetic acid and acetonitrile with 0.01% acetic acid were ultimately chosen for the better separation and steady baseline. Different column temperatures and flow rates were investigated, and 28 °C of column temperature and 1.0 mL/min were separately selected considering both the chromatographic separation and system pressure. While the injection volume was less than 15 µL, several low content peaks would disappear. Thus, 20 µL was finally chosen as the injection volume. The gradient program was optimized, and the final adopted gradient program was described in Experimental section.

### Method validation of fingerprinting

The optimized method was validated in terms of precision, repeatability, and stability tests. The precision was determined by loading the same sample solution S1 for six times consecutively. The RSD of common peaks retention times (t_R_) were less than 0.2%, and the RSD of peak area with area more than 1% of the total peak area (PA) were less than 1.0%. The similarities were calculated by comparing the chromatographic fingerprint with that of the first test, and the similarities were all 1.000. The repeatability was investigated by analyzing six independently prepared solutions of the same sample (S1). The RSD of t_R_ and PA were less than 0.5% and 2.0%, respectively. The similarities were calculated by comparing the chromatographic fingerprint with that of the first test, and the similarities were all 1.000. The stability was evaluated by analyzing a single sample solution (S1) stored at room temperature for 0, 2, 6, 12, 18, and 24 h. RSDs of t_R_ and PA were less than 1.6% and 0.8%, respectively. The similarities were calculated by comparing the chromatographic fingerprint with that of one test, and all similarities were 1.000. All results above indicated that this analysis method was able to meet the requirements of the fingerprinting analysis.

### Similarity evaluation of XST by chromatographic fingerprinting

**Figure 5:**
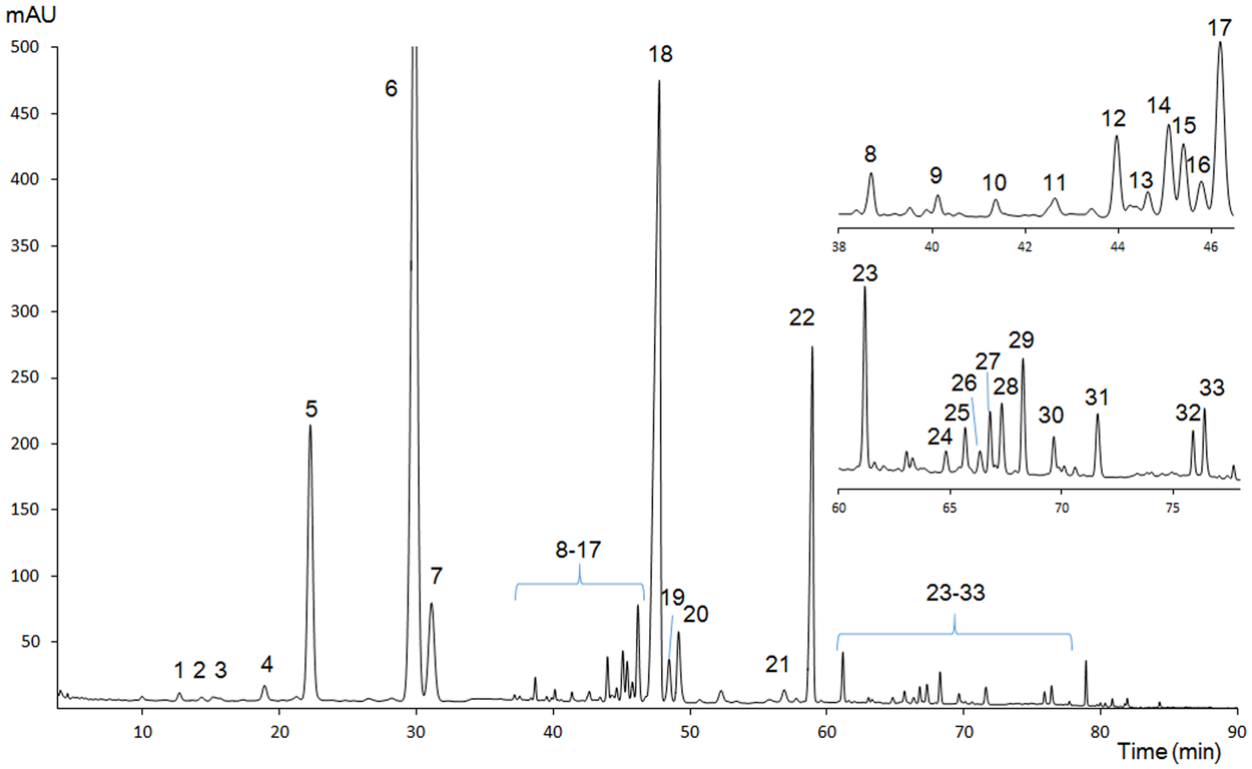
HPLC fingerprint chromatogram of XST. 1. 20-O-Glucoginsenoside Rf, 2. Yixinoside A/Gypenoside XLIV, 3. Notoginsenoside N, 4. Ginsenoside M_6-a_, 5. NG-R_1_*, 6. G-Rg_1_*, 7. G-Re*, 8. Vinaginsenoside R_20_, 9. 3β,12β,20S-trihydroxydammar-(E)-24-ene-6-O-β-D-xylopyranosyl-(1 → 6)-β-D-glucopyranoside, 10. Notoginsenoside A, 11, Notoginsenoside D/Notoginsenoside T, 12. Notoginsenoside Fa, 13. Notoginsenoside U, 14. Chikusetsusaponin VI, 15. Chikusetsusaponin L_5_/Notoginsenoside I, 16. 5,6-Didehydroginsenoside Rb_1_, 17. 20(S)-NG-R_2_*, 18. G-Rb_1_*, 19. G-Rg_2_*, 20. G-Rh_1_*/Ginsenoside Ra_1_, 21. G-F_1_*, 22. G-Rd*, 23. Gy-XVII*, 24. NG-T_5_*, 25. 20D-NG-R_2_*/Ginsenoside Rg_6_, 26. G-F_4_*, 27. G-F_2_*, 28. G-Rk_3_*, 29. G-Rh_4_*, 30. 20(S)-G-Rg_3_*, 31. Falcarindiol, 32. G-Rk_1_*, 33. G-Rg_5_*. (*Identified with reference standards).

The typical chromatogram of XST was shown in Fig. 5. Thirty-three common peaks were identified by referring to the result in the first step of this strategy. Eight batches of XST were collected for the batch-to-batch quality consistency evaluation. All similarities of 8 samples of XST were calculated by comparing the sample chromatographic fingerprint with the mean chromatogram. The results of similarities were shown in Table S4. All the similarities were higher than 0.996, indicating that samples shared a similar chromatographic pattern and there was a good batch-to-batch quality consistency between selected batches of XST.

### Constituents origins and variations during manufacturing process

**Figure 6:**
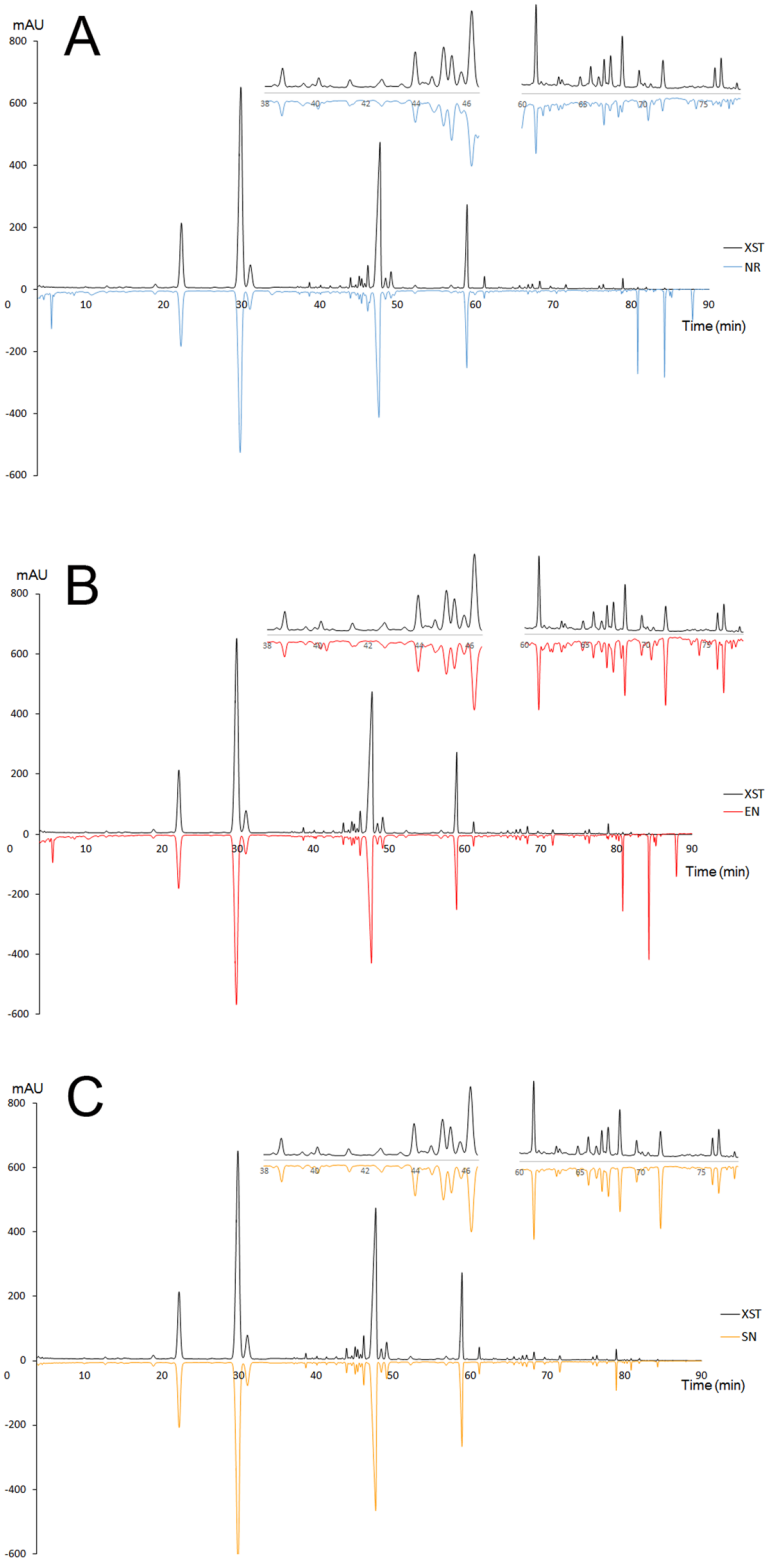
Constituents origins and variations during manufacturing process. (**A**) XST vs. NR, (**B**) XST vs. EN and (**C**) XST vs. SN.

CMC is important information required for new drug application in many countries. Besides botanical drug substance, manufacturing process is another essential portion of CMC information. A series of process control studies have been performed by our group using process analytical technology^31–33^. After identification of BCM in XST, herein, chemical delivery of BCM and other constituents was tentatively investigated from raw materials to final products of XST. There are four main manufacturing procedures of XST. First, the raw materials of *Notoginseng Rhizoma* (NR) are extracted by 70% ethanol solution to produce the extracts of *Notoginseng Rhizoma* (EN); Then, the EN are purified to obtain the total saponins of *Notoginseng Rhizoma* (SN); The SN undergoes a series of processes to produce the final XST product. The fingerprints of NR, EN, SN and XST were analyzed and compared. The origins of constituents in XST product and their variation during the manufacturing process were illustrated in Fig. 6. The proportions variations of BCM (NG-R_1_, G-Rg_1_, G-Re, G-Rb_1_ and G-Rd) were shown in Fig. S1. The contents of NG-R_1_, G-Rg_1_, G-Re and G-Rb_1_ did not fluctuate evidently, however G-Rd exhibited a certain degree of declining trend during the manufacturing process. The proportion of the peaks for the rest 28 common peaks were presented as the ratio of the peak area to the total peak area. As shown in Fig. S2, only the proportion of 20D-NG-R_2_/G-Rg_6_, NG-T_5_, G-F_4_, G-Rk_3_, G-Rh_4_, 20(S)-G-Rg_3_, G-Rk_1_ and G-Rg_5_ fluctuated evidently, which increased significantly after extraction and concentration (as shown in EN), then displayed a downward trend during later processes (as shown in SN and XST). It was suggested that increased ginsenosides might be originated from the degradation of the main saponins during the concentration process.

**Figure 7:**
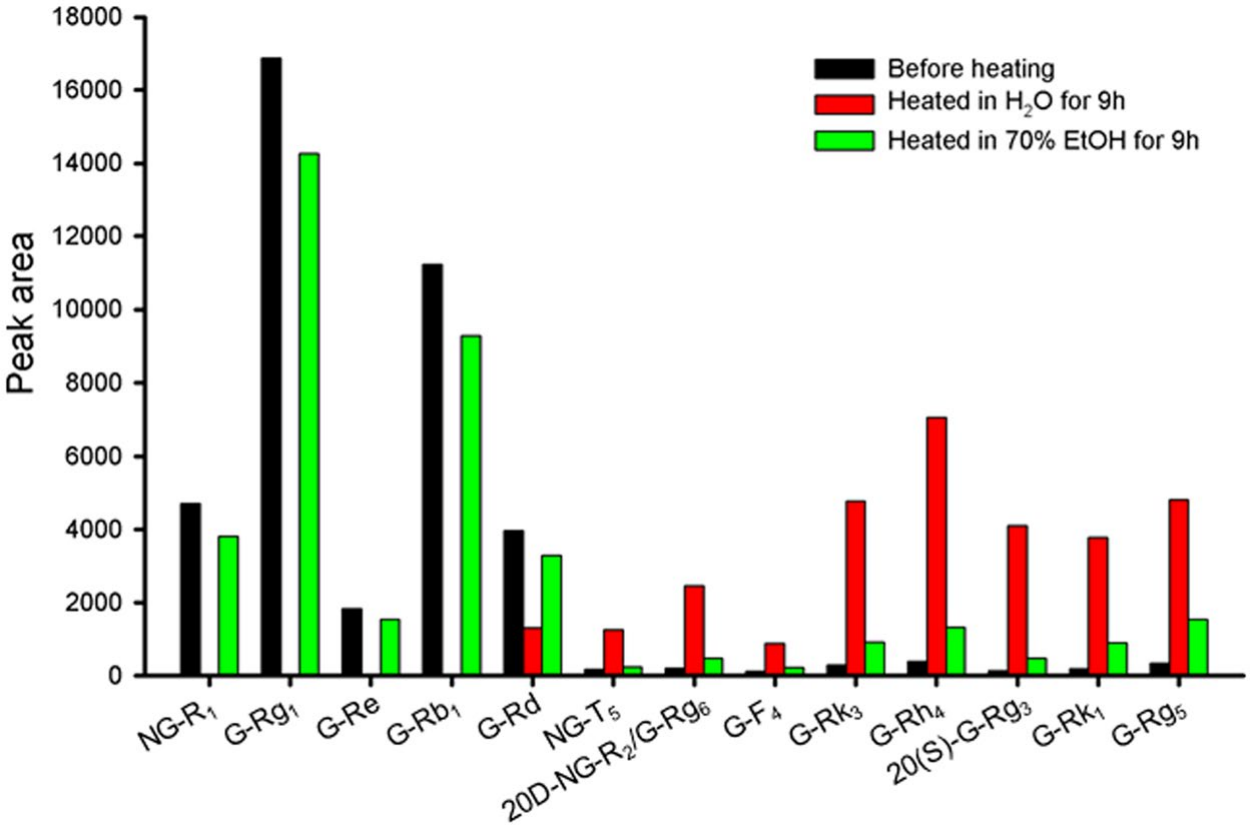
Proportions variations of constituents in accelerated degradation experiment.

During the concentration process, the concentration of ethanol in the concentrated solution would decrease, which might be the cause of the degradation. Thus, an accelerated degradation experiment was carried out using solutions with different proportions of ethanol to verify this hypothesis. Concretely, the EN sample was heated at 105 ◦C for 9 h in aqueous solution (simulating an extreme condition during concentration process) or 70% ethanol solution (simulating the beginning of concentration process), separately. After heated in aqueous solution, the amount of five main saponins decreased significantly, as shown in Fig. 7. Specifically, almost all of NG-R_1_, G-Rg_1_, G-Re and G-Rb_1_, as well as most of G-Rd, degraded. In the meantime, considerable 20D-NG-R_2_/G-Rg_6_, NG-T_5_, G-F_4_, G-Rk_3_, G-Rh_4_, 20(S)-G-Rg_3_, G-Rk_1_ and G-Rg_5_ were generated. However, in 70% ethanol solution, the variations of saponins in quantity were relatively mild. Thus, the proportion variation of the saponins in EN shown in Figs S1 and S2, might be mainly caused by the change of ethanol proportion during concentration. In the concentration process, the ethanol proportion decreased as the evaporation of solution. When the ethanol concentration was low, sustained heating would advance the degradation of the main saponins. Results from accelerated degradation experiment indicated that it is important to perform simultaneous measurement of the BCM and minor common peaks through fingerprint for ensuring the quality consistency of final products.

Through this part of study, it could be seen that the contents of BCM and other constituents in XST varied during the manufacturing process, and further study about process control of XST would be performed in the future.

## Conclusion

A novel efficacy and consistency oriented strategy for quality assessment of botanical drugs was proposed and applied to XST to confirm its utility. After thorough chemical characterization of XST, its BCM, i.e., NG-R_1_, G-Rg_1_, G-Re, G-Rb_1_ and G-Rd., were identified using the techniques of pharmaceutical informatics. Combined BCM demonstrated efficacy equivalence with XST *in vivo*, which suggested they could serve as a group of rational chemo-markers to ensure the efficacy of XST. The total content of BCM represent more than 85% of the solid mass of XST, as shown in Fig. S3, and both the content of identified constituents and identified constituents presented in the chemical fingerprint was more than 95% of the solid content of XST. Current study not only satisfies the criteria in this *Requirements* of CFDA, but also might provide a workflow for CMC research required for botanical drug application.

## Experimental

### Chemicals, materials and instrumentation

HPLC-grade acetonitrile and methanol were purchased from Merck (Darmstadt, Germany). HPLC-grade formic acid and acetic acid was purchased from Roe Scientific Inc. Water was purified by Milli-Q system (Millipore, Bedford, MA, USA). Chloralic hydrate was purchased from Kemi’ou chemical reagent Inc. (Tianjin, China). 2,3,5-Triphenyltetrazolium chloride (TTC) was purchased from Tianjin Guangfu Chemicals (Tianjin, China). All other chemicals and solvents were of analytical grade.

Notoginsenoside R_1_ (NG-R_1_), ginsenoside Rg_1_ (G-Rg_1_), ginsenoside Re (G-Re), ginsenoside Rb_1_ (G-Rb_1_), ginsenoside Rg_2_ (G-Rg_2_), 20(S)-ginsenoside Rh_1_ (20(S)-G-Rh_1_), 20(R)-ginsenoside Rh_1_ (20(R)-G-Rh_1_), ginsenoside Rb_2_ (G-Rb_2_), ginsenoside F_1_ (G-F_1_), ginsenoside F_2_ (G-F_2_), ginsenoside Rd (G-Rd), 20(S)-ginsenoside Rg_3_ (20(S)-G-Rg_3_), 20(R)-ginsenoside Rg_3_ (20(R)-G-Rg_3_) and gypenoside XVII (Gy-XVII) were purchased from Ronghe Pharmaceutical Technology Development Co. Ltd. (Shanghai, China). Ginsenoside Rk_1_ (G-Rk_1_), ginsenoside Rg_5_ (G-Rg_5_), ginsenoside F_4_ (G-F_4_), ginsenoside Rk_3_ (G-Rk_3_), notoginsenoside Rh_4_ (NG-Rh_4_), 20(S)-notoginsenoside R_2_ (20(S)-NG-R_2_), ginsenoside T_5_ (G-T_5_) and 3β,12β-dihydroxydammar-(E)-20(22),24-diene-6-O-β-D-xylopyranosyl-(1 → 2)-β-D-glucopyranoside (20,22-dehydranotoginsenoside R_2_, 20D-NG-R_2_) were purified in Pharmaceutical Informatics Institute, Zhejiang University. XST lyophilized powder, raw materials of *Notoginseng Rhizoma* (NR), extracts of *Notoginseng Rhizoma* (EN) and the total saponins of *Notoginseng Rhizoma* (SN) were obtained from Heilongjiang ZBD Pharmaceutical Co., Ltd. (Heilongjiang, China).

### Characterization of the constituents in XST

About 400 mg XST lyophilized powder was dissolved in 50% methanol-H_2_O solvent to obtain a XST solution with a concentration of 100 mg/mL. In order to decrease the complexity and enrich the minor constituents, the XST solution was firstly dissected into multiple fractions by preparative HPLC system (Agilent, Waldbronn, Germany), and then HPLC-MS^n^ was employed for the constituents characterization of these fractions separately. Fractions of XST were separated by preparative HPLC with mobile phase consisted of H_2_O (phase A) and acetonitrile (phase B) at a flow rate of 10 mL/min. The elute gradient was as follows: 0–30 min, 19–21% B; 30–40 min, 21–28% B; 40–55 min, 28–33% B; 55–85 min, 33–45% B; 85–95 min, 45–70% B; 95–105 min, 70–95% B. The fractions were collected as follows: Fr. 1, 4.0–10.0 min; Fr. 2, 10.0–30.3 min; Fr. 3, 30.3–32.9 min; Fr. 4, 32.9–38.4 min; Fr. 5, 38.4–40.8 min; Fr. 6, 40.8–50.4 min; Fr. 7, 50.4–57.6 min; Fr. 8, 57.6–60.5 min; Fr. 9, 60.5–65.6 min; Fr. 10, 65.6–107.4 min. The fractions were then analyzed by HPLC-MS^n^ and the elute gradients of the fractions varied owing to the difference polarity among the fractions. The MS and MS^n^ analyses were carried out using Finnigan LCQ Deca XP^plus^ ion trap mass spectrometer (Thermo Finnigan, San Jose, CA, USA) coupled to an Agilent 1100 HPLC system (Agilent, Waldbronn, Germany). Ultrahigh pure helium (He) and high purity nitrogen (N_2_) were used as collision and nebulizing gases, respectively. The mass spectra were acquired in the negative ion mode. An Agilent Zorbax SB-C_18_ column (4.6 mm × 250 mm, 5 μm) (Agilent, USA) was used for separation. The mobile phase comprises water containing 0.01% acetic acid (solvent A) and acetonitrile containing 0.01% acetic acid (solvent B).

### Establishment of BCM identification method based on Adjusted Efficacy Score

For treating CCVD, botanical drugs usually exhibited therapeutic effects from several aspects. In this study, the anti-CCVD effects of the constituents in XST were divided into seven aspects, including myocardial protection^34, 35^, vascular protection^36, 37^, anticoagulation^29, 38^, antihypertension^38–41^, anti-inflammation^29, 42–44^, anti-oxidation^45–47^, and improvement of carbohydrate and lipid metabolism^38–40^. By manually text-mining the published literatures in Pubmed, the anti-CCVD effects of these constituents in XST were summarized. The anti-CCVD effect was evaluated by Reverse Rate (RR)^48, 49^, which was calculated by equation (1).

1 $$RR=\frac{{M}_{j}-{T}_{j}}{{M}_{j}-{N}_{j}}$$

where *N*_*j*_, *M*_*j*_ and *T*_*j*_ indicate the performances of normal, model and constituent treated group in report *j*.

Efficacy Score (ES) was developed to indicate the integrated anti-CCVD effect of these constituents, which was calculated by equation (2).

2 $$ES(a)=\frac{1}{m}\sum _{i=1}^{m}(\frac{1}{n}\sum _{j=1}^{n}R{R}_{ij})$$

where *RR*_*ij*_ is the reverse rate of a constituent in one report, *n* is the number of reports about an aspect of anti-CCVD effects, *m* is the number of anti-CCVD effect aspects which is 7 in this study.

The content of the constituent in botanical drugs was also an important factor to be considered, which influenced the role the constituent played in botanical drugs. Adjusted ES (AES) was developed to represent the importance of the constituent in botanical drugs for the therapeutic efficacy, which was calculated by equation (3).

3 $$AES(a)=\frac{c(a)\times ES(a)}{{\sum }_{a=1}^{w}[c(a)\times ES(a)]}\times 100 \% $$

where *c*(*a*) is the content of constituent *a*, and *w* is the number of constituents quantified.

### Effect estimation of potential BCM on myocardial ischemia

To further verify the potential BCM of XST, the saponins were combined with proportions in XST to investigate whether they were bioactive equivalent to XST *in vivo*. The classic rat model of myocardial infarction (MI) was utilized^50^ and performed by Prof. Zhuanyou Zhao’s lab (Tianjin Institute of Pharmaceutical Research), which is an Association for Assessment and Accreditation of Laboratory Animal Care International approved facility. All experiments were carried out with the approval of Institutional Animal Ethical Committee of Tianjin Centre for Drug Safety Assessment (Tianjin, China), and all methods were performed in accordance with the relevant guidelines and regulations.

Male Sprague–Dawley rats (230–295 g) were obtained from Vital River Laboratory Animal Technology Co. Ltd. (Beijing, China). The Institutional Animal Ethical Committee of Tianjin Centre for Drug Safety Assessment (Tianjin, China) approved all the animal procedures.

MI was produced by occlusion of the left anterior descending coronary artery (LAD). The model construction was confirmed if the ST-segment elevation in electrocardiogram (ECG) was more than 0.2 mV after 15 min. Sham-operated rats underwent the same procedure but without LAD occlusion. Rats were divided to six groups: Sham group (vehicle, *i.v*.), Model group (vehicle, i.v.), XSTH group (150 mg/kg XST, *i.v*.), XSTL group (100 mg/kg XST, *i.v*.), XST5H group (five main saponins, i.e., NG-R_1_, G-Rg_1_, G-Re, G-Rb_1_ and G-Rd, with related proportion and dose in 150 mg/kg XST, *i.v*.) and XST5L group (five main saponins, i.e., NG-R_1_, G-Rg_1_, G-Re, G-Rb_1_ and G-Rd, with related proportion and dose in 100 mg/kg XST, *i.v*.), with n = 10 in each group. XST, XST5 and vehicle were administrated at 15 min after LAD ligation.

After 24 h-treatment, blood samples were taken from the abdominal aorta. The serum was separated to evaluate the myocardial marker enzymes including creatine kinase (CK), MB isoenzyme of creatine kinase (CK-MB) and lactate dehydrogenase (LDH).

After the blood was taken, 1 mL of 1% Evans blue was injected into the femoral vein, and the left ventricle was removed 1 min later. After washed with physiological saline and frozen in −20 °C, the left ventricles were sliced transversely into 5 slices. The sliced sections were then stained with 1% TTC for 10 min to delineate the infracted myocardium. Infarct size was measured by MP150 biomedical data acquisition system (Biopac Systems Inc, USA), and results were expressed as the percentage of the infarct size to the entire left ventricle.

### Content determination and HPLC fingerprinting

#### Preparation of samples

XST lyophilized powder (50 mg) was accurately weighed, transferred into calibrated flasks, extracted for 5 min with 4 mL of 50% methanol in an ultrasonic bath, and then diluted to a 5 mL solution accurately. After centrifuged at 10000 rpm for 10 min, the supernatant was taken for further analysis.

EN and SN also prepared in a similar manner to obtain the sample solutions. NR was firstly extracted with 70% methanol in an ultrasonic bath for 1 h, then concentrated to remove the solution. The extracts of NR were used to prepare the sample solutions of NR.

#### Chromatography conditions for quantitative determination and HPLC fingerprinting

Chromatographic separations were performed on an Agilent 1100 HPLC system. HPLC separation was carried out on an Agilent Zorbax SB-C_18_ column (4.6 mm × 250 mm, 5 μm) with the column temperature set to 28 °C. Aqueous 0.01% acetic acid (phase A) and 0.01% acetic acid in acetonitrile (phase B) were selected as mobile phase solvents. The flow rate was 1 mL/min. The gradient program for quantitative analysis was as follows: 0–30 min, 19–21% B; 30–35 min, 21–28% B; 35–41 min, 28–32% B; 41–50 min, 32% B; 50–55 min, 32–42% B; 55–60 min, 42% B. For the quantitative analysis, the injection volume was 5 µL. HPLC fingerprinting gradient program was as follows: 0–30 min, 19–21% B; 30–35 min, 21–28% B; 35–41 min, 28–32% B; 41–52 min, 32% B; 52–70 min, 32–53% B; 70–80 min, 53–90% B; 80–90 min, 90% B. The injection volume was 20 µL.

#### Similarity evaluation

The similarity analysis was performed by professional software named Similarity Evaluation System for Chromatographic Fingerprint of Traditional Chinese Medicine (Version 2004 A, Chinese Pharmacopoeia Commission).

## Electronic supplementary material

Supplementary information
