# Supplementary material for: A Bioactive Chemical Markers Based Strategy for Quality Assessment of Botanical Drugs: Xuesaitong Injection as a Case Study
Source: Sci Rep. 2017 May 25;7:2410. doi: 10.1038/s41598-017-02305-y (PMC5445085; doi:10.1038/s41598-017-02305-y)
Supplement: Supplementary file 1 — Supplementary information [file 41598_2017_2305_MOESM1_ESM.pdf]

Supplementary Information

A Bioactive Chemical Markers Based Strategy for Quality Assessment of Botanical Drugs: Xuesaitong Injection as a Case Study

Zhenzhong Yang[1], Qing Shao[1], Zhiwei Ge[1], Ni Ai[1], Xiaoping Zhao[2], Xiaohui Fan[1,*]

[1]*Pharmaceutical Informatics Institute, College of Pharmaceutical Sciences, Zhejiang University, Hangzhou 310058, China.*

[2]*College of Preclinical Medicine, Zhejiang Chinese Medical University,Hangzhou 310053, China.*

* *Author to whom correspondence should be addressed; E-Mail: fanxh@zju.edu.cn; Tel.: +86-571-88208596; Fax: +86-571-88208426.*

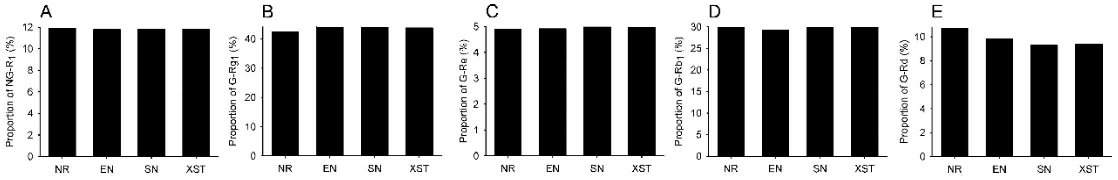

Fig. S1 The proportions variations of the five main saponins. (A) NG-R_1, (B) G-Rg_1, (C) G-Re, (D) G-Rb_1 and (E) G-Rd.

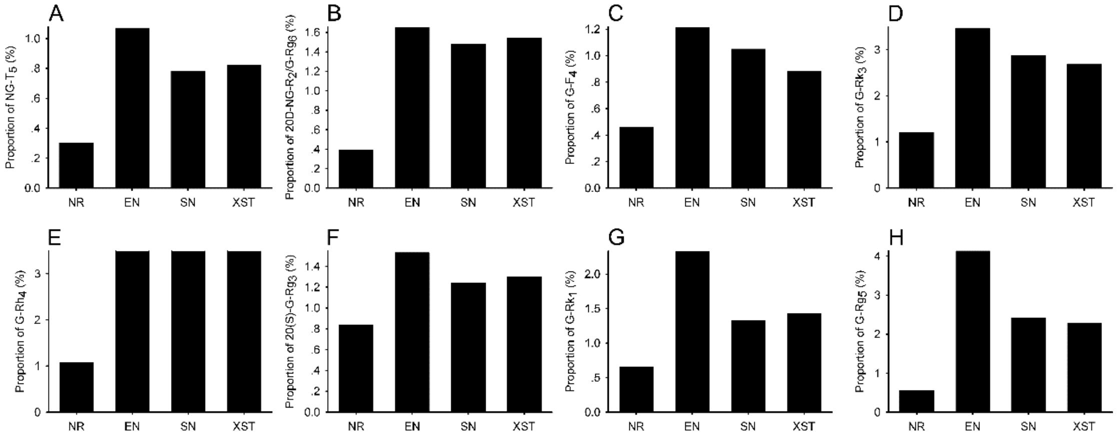

Fig. S2 The proportions variations of the minor saponins. (A) 20D-NG-R_2/G-Rg_6, (B) NG-T_5, (C) G-F_4, (D) G-Rk_3, (E) G-Rh_4, (F) 20(S)-G-Rg_3, (G) G-Rk_1 and (H) G-Rg_5.

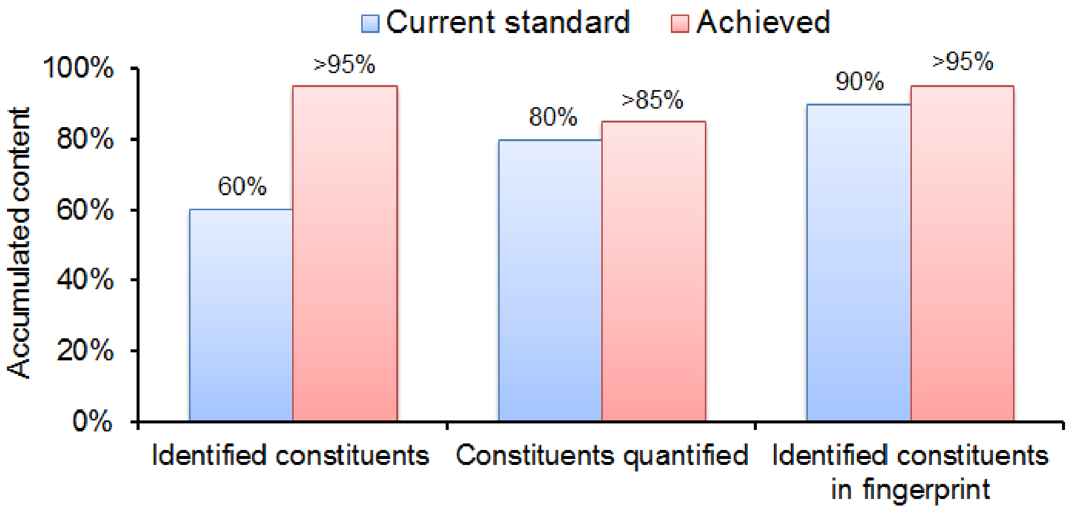

Fig. S3 Current standard of CFDA and achievements in the quality assessment of XST.

Table S1. Characterization of constituents in XST by LC-IT/MS^n

Compounds No.	t_R (min)	$[M-H]^-$ (*m/z*)	MS^n data (*m/z*)	Characterization of compounds
1-1	31.9	947	785, 491	Notoginsenoside H
1-2	37.3	961	799, 653, 491	Majoroside F_6
1-3	37.7	961	799, 653, 491	Majoroside F_5
2-1	11.3	1093	799, 637, 475	2α,3β,12β,20S-tetrahydroxydammar-24-ene-3-O-[β-D-glucopyranosyl(1→4)-β-D-glucopyranosyl]-20-O-[β-D-xylopyranosyl-(1→6)-β-D-glucopyranoside]
2-2	13.2	1093	799, 637, 475	Floralginsenoside P
2-3	14.3	961	799, 637, 475	20-O-Glucoginsenoside Rf
2-4	15.4	1093	799, 637, 475	Gypenoside LXIX
2-5	15.8	785	553, 491	Notoginsenoside Rw_2
2-6	16.1	1107	799, 637, 475	Yixinoside A
2-7	16.2	961	799, 637, 475	Gypenoside XLIV
2-8	17.0	1107	799, 637, 475	Gypenoside XLIII
2-9	17.3	961		Notoginsenoside N
2-10	18.8	1107	799, 637, 475	Yesanchinoside E
2-11	19.3	785	653, 491	Vinaginsenoside R_{11}
2-12	20.4	1093	799, 637, 475	Floranotoginsenoside A
2-13	20.6	931	799, 637, 475	Gypenoside LVII or Gypenoside LXIV
2-14	21.0	961	799, 637, 475	Ginsenoside $M_{6\text{-}a}$
2-15	21.8	1093	799, 637, 475	Floranotoginsenoside D
2-16	21.9	785	653, 491, 491	Pseudoginsenoside RT_2
2-17	23.0	785	653, 491, 491	Majonoside R_2
2-18	25.4	961	799, 637, 475	Notoginsenoside M

Compounds No.	t_R (min)	$[M-H]^-$ (*m/z*)	MS^n data (*m/z*)	Characterization of compounds
3-1	25.0	931	799, 637, 475	Notoginsenoside R_1*
4-1	25.9	799	653, 491, 491	24(S)-Pseudoginsenoside F_{11}
4-2	26.4	961	799, 637, 475	Ginsenoside Re_3
4-3	27.7	799	653, 491, 491	Pseudoginsenoside F_{11}
4-4	28.7	945	637, 475	Gypenoside XLV
4-5	30.2	945	783, 637, 475	Gypenoside LXXII
4-6	31.2	799	637, 475, 475	Ginsenoside Ia
4-7	31.9	961	799, 637, 475	Notoginsenoside R_3
4-8	32.8	1141	1105, 943	Notoginsenoside K or C
4-9	33.2	1121	959, 797, 473	Quinquenoside IV
5-1	34.5	799	637, 475	Ginsenoside Rg_1*
5-2	35.3	945	799, 637, 475	Ginsenoside Re*
5-3	36.4	1141	979, 817, 493	Dihydroxyginsenoside Rb_1
6-1	35.5	1033	901, 769, 475	Succulentoside D
6-2	35.9	799	637, 475, 475	(20R)-Ginsenoside Rg_1
6-3	36.4	931	799, 637, 475	Notoginsenoside Fp_1
6-4	37.4	1123	781	Gypenoside XLII
6-5	37.9	901	769, 607, 475	Notoginsenoside Rw_1
6-6	38.0	1107	943, 781, 457	Gypenoside XIX
6-7	38.1	799	637, 475, 475	Ginsenoside Rf
6-8	38.6	901	769, 637, 475	Chikusetsusaponin L_5
6-9	38.8	959	797, 635, 473	Vinaginsenoside R_{20}
6-10	38.8	769	607, 475	Pseudoginsenoside RT_3
6-11	39.3	1123	961	Gypenoside XLVII

Compounds No.	t_R (min)	$[M-H]^-$ (*m/z*)	MS^n data (*m/z*)	Characterization of compounds
6-12	39.7	1121	959, 779, 473	Notoginsenoside B
6-13	40.1	769	637, 475	3β,12β,20S-trihydroxydammar-(E)-24-ene-6-O-β-D-xylopyranosyl-(1→6)-β-D-glucopyranoside
6-14	40.3	1107	943, 781, 0	Luperoside G
6-15	40.8	931	799, 637, 475	Ginsenoside F_6
6-16	41.8	1123	961	Notoginsenoside A
6-17	42.2	1239	1107, 945	β-D-Glucopyranoside, (3β,12β)-3-[(2-O-β-D-glucopyranosyl-β-D-glucopyranosyl)oxy]-12-hydroxydammar-24-en-20-yl O-β-D-glucopyranosyl-(1→4)-O-[β-D-xylopyranosyl-(1→6)]- (9CI)
6-18	42.2	1371	1239	Notoginsenoside D or T
6-19	42.5	1107	943, 781, 457	Luperoside H
6-20	43.0	1269	1107, 945, 459	Ginsenoside RA_0
6-21	43.4	1269	1107, 945, 459	Quinquenoside V
6-22	43.5	1107	945, 783, 459	Ginsenoside b_1
6-23	43.8	1123	961, 475	Epoxyginsenoside Rb_1
6-24	44.1	1239	1107, 945, 459	Notoginsenoside R_4
6-25	46.8	1107	945, 783, 459	Ginsenoside Rb_1*
7-1	44.0	799		Majoroside F_4
7-2	44.6	1239		Notoginsenoside Fa
7-3	44.9	799	637, 475	Notoginsenoside U
7-4	46.7	1105		5,6-Didehydroginsenoside Rb_1
7-5	47.0	769		20(S)-Notoginsenoside R_2*
7-6	47.5	1239	1107, 459	Chikusetsusaponin VI
7-7	47.6	901	769, 637	Chikusetsusaponin L_5

Compounds No.	t_R (min)	$[M-H]^-$ (*m*/*z*)	MS^n data (*m*/*z*)	Characterization of compounds
7-8	47.9	1091	929, 767	Notoginsenoside I
8-1	48.9	783	637, 475	Ginsenoside Rg_2*
8-2	49.2	1209	1077, 945, 459	Ginsenoside Ra_1
8-3	49.6	637	475, 391	20(S)-Ginsenoside Rh_1*
8-4	50.6	1209	1077, 945, 459	Ginsenoside Ra_2
8-5	51.1	637	475	20(R)-Ginsenoside Rh_1 *
8-6	51.7	1077	945, 783, 459	Notoginsenoside L
8-7	52.5	1077	945, 783, 459	Ginsenoside Rb_2*
9-1	52.7	1077	945, 783, 459	Ginsenoside Rc
9-2	53.3	797	635, 473	Gypenoside XL
9-3	55.4	797	635, 605, 473	Gypenoside XXXIII
9-4	56.1	637	475, 391	Ginsenoside F_1*
9-5	56.9	945	783, 621, 459	Ginsenoside Rd*
9-6	57.8	1077	945, 783, 459	Vinaginsenoside R_7
9-7	58.1	945	783, 621, 459	Gypenoside XVII*
10-1	58.1	915	753	Vinaginsenoside R_{17}
10-2	58.8	915	783, 621, 459	Notoginsenoside Fd
10-3	60.4	751	619, 499	Notoginsenoside T_5*
10-4	60.9	929	783, 459	Gypenoside X
10-5	60.9	765	619, 457	Ginsenoside Rg_6
10-6	61.0	751	619, 457	3β,12β-dihydroxydammar-(E)-20(22),24-diene-6-O-β-D-xylopyranosyl-(1→2)-β-D-glucopyranoside*
10-7	61.5	765	619, 457	Ginsenoside F_4*
10-8	61.8	783	621, 459	Ginsenoside F_2*

Compounds No.	t_R (min)	$[M-H]^-$ (*m*/*z*)	MS^n data (*m*/*z*)	Characterization of compounds
10-9	62.1	915	783, 621, 459	Notoginsenoside Ft_1
10-10	62.5	619		Ginsenoside Rk_3*
10-11	63.4	619		Ginsenoside Rh_4*
10-12	64.1	783	621, 459	20(S)-Ginsenoside Rg_3*
10-13	64.6	783	621, 459	20(R)-Ginsenoside Rg_3*
10-14	66.3	261[#]		Falcarindiol
10-15	70.2	765	603, 473	Ginsenoside Rk_1*
10-16	70.7	765	603, 473	Ginsenoside Rg_5*

* Identified with reference compounds.

[#] Obtained in positive ion mode.

Table S2. Reverse Rate (RR) of the constituents in XST by manually text-mining the published literatures in Pubmed.

Constituent	RR_{ave}							*ES*	References
	**Myocardial protection**	**Vascular protection**	**Anticoagulation**	**Anti-hypertension**	**Anti-inflammation**	**Anti-oxidation**	**Improvement of carbohydrate and lipid metabolism**		
Ginsenoside Rb_1	0.47	0.89	-	0.70	0.52	0.80	0.34	0.53	[1-20]
Ginsenoside Rg_1	0.36	0.73	0.87	-	0.66	1.33	1.80	0.70	[21-35]
Ginsenoside Rd	0.36	-	-	0.14	0.83	0.70	-	0.29	[36-41]
Ginsenoside Re	0.74	0.64	-	-	0.65	0.77	0.69	0.50	[42-56]
Notoginsenoside R_1	0.56	0.61	-	0.92	0.53	0.53	0.27	0.49	[46, 57-64]
Ginsenoside F_1	-	-	-	-	0.46	0.45	-	0.13	[65]
Ginsenoside F_2	-	-	-	-	-	-	0.88	0.13	[66]
Ginsenoside Rb_2	-	-	-	-	0.48	0.70	1.13	0.33	[48, 67-71]
Ginsenoside Rg_5	-	1.33	-	-	0.59	0.82	-	0.39	[72-77]
Ginsenoside Rg_2	0.50	0.85	-	-	-	0.86	-	0.32	[78, 79]
Ginsenoside Rh_1	0.48	0.45	-	-	0.66	0.61	0.65	0.41	[65, 80-84]
Ginsenoside Rh_4	-	-	-	-	0.29	-	-	0.04	[85]
Ginsenoside Rk_1	-	0.81	-	-	0.77	0.30	-	0.27	[75, 77, 86]
Ginsenoside Rk_3	0.62	-	-	-	0.63	-	-	0.18	[87, 88]
Gypenoside XVII	-	-	-	-	-	0.99	-	0.14	[89]
Notoginsenoside R_2	-	-	-	-	-	0.72	-	0.10	[90]

Table S3. Contents of bioactive chemical markers in 8 batches of XST.

Batch No.	Contents (%)					
	Notoginsenoside R1	Ginsenoside Rg1	Ginsenoside Re	Ginsenoside Rb1	Ginsenoside Rd	Bioactive chemical markers
S1	9.348±0.04	34.65±0.18	4.980±0.04	31.67±0.18	7.181±0.05	87.83±0.45
S2	9.127±0.05	33.87±0.37	4.610±0.07	30.82±0.36	6.825±0.07	85.25±0.88
S3	9.209±0.03	34.35±0.23	4.657±0.11	31.10±0.24	6.813±0.05	86.13±0.63
S4	8.925±0.07	33.84±0.13	4.450±0.06	30.86±0.12	6.859±0.03	84.93±0.26
S5	10.01±0.15	34.50±0.44	4.457±0.07	33.09±0.42	7.726±0.08	89.78±0.96
S6	9.176±0.09	33.68±0.65	4.673±0.23	32.84±0.50	7.156±0.08	87.52±1.50
S7	9.183±0.11	33.88±0.27	4.657±0.05	31.83±0.05	7.132±0.07	86.68±0.45
S8	9.215±0.06	32.14±0.06	4.352±0.07	35.22±0.10	7.388±0.02	88.32±0.26

Table S4. Similarity of 8 batches of XST.

Sample No.	Similarity
S1	1.000
S2	0.999
S3	0.999
S4	1.000
S5	1.000
S6	0.999
S7	0.999
S8	0.997

References:

1. Guan, L., Li, W. & Liu, Z. Effect of ginsenoside-Rb1 on cardiomyocyte apoptosis after ischemia and reperfusion in rats. *J Huazhong Univ Sci Technolog Med Sci***22**, 212-5 (2002).
2. Wang, Z., Li, M., Wu, W.K., Tan, H.M. & Geng, D.F. Ginsenoside Rb1 preconditioning protects against myocardial infarction after regional ischemia and reperfusion by activation of phosphatidylinositol-3-kinase signal transduction. *Cardiovasc Drugs Ther***22**, 443-52 (2008).
3. Li, G., Qian, W. & Zhao, C. Analyzing the anti-ischemia-reperfusion injury effects of ginsenoside Rb1 mediated through the inhibition of p38alpha MAPK. *Can J Physiol Pharmacol*, 1-7 (2015).
4. Xia, R. et al. Ginsenoside Rb1 preconditioning enhances eNOS expression and attenuates myocardial ischemia/reperfusion injury in diabetic rats. *J Biomed Biotechnol***2011**, 767930 (2011).
5. Yan, X. et al. Ginsenoside rb1 protects neonatal rat cardiomyocytes from hypoxia/ischemia induced apoptosis and inhibits activation of the mitochondrial apoptotic pathway. *Evid Based Complement Alternat Med***2014**, 149195 (2014).
6. Kong, H.L. et al. Anti-hypoxic effect of ginsenoside Rbl on neonatal rat cardiomyocytes is mediated through the specific activation of glucose transporter-4 ex vivo. *Acta Pharmacol Sin***30**, 396-403 (2009).
7. Wang, X.F. et al. Ginsenoside rb1 reduces isoproterenol-induced cardiomyocytes apoptosis in vitro and in vivo. *Evid Based Complement Alternat Med***2013**, 454389 (2013).
8. Song, Z. et al. Ginsenoside Rb1 prevents H2O2-induced HUVEC senescence by stimulating sirtuin-1 pathway. *PLoS One***9**, e112699 (2014).
9. Zhou, W. et al. Ginsenoside Rb1 blocks homocysteine-induced endothelial dysfunction in porcine coronary arteries. *J Vasc Surg***41**, 861-8 (2005).
10. Lan, T.H. et al. Ginsenoside Rb1 prevents homocysteine-induced endothelial dysfunction via PI3K/Akt activation and PKC inhibition. *Biochem Pharmacol***82**, 148-55 (2011).
11. Ohashi, R. et al. Effects of homocysteine and ginsenoside Rb1 on endothelial proliferation and superoxide anion production. *J Surg Res***133**, 89-94 (2006).

12. He, F., Guo, R., Wu, S.L., Sun, M. & Li, M. Protective effects of ginsenoside Rb1 on human umbilical vein endothelial cells in vitro. *J Cardiovasc Pharmacol* **50**, 314-20 (2007).
13. Li, Q.Y. et al. Ginsenoside Rb1 inhibits proliferation and inflammatory responses in rat aortic smooth muscle cells. *J Agric Food Chem* **59**, 6312-8 (2011).
14. Lin, X.H., Hong, H.S., Zou, G.R. & Chen, L.L. Upregulation of TRPC1/6 may be involved in arterial remodeling in rat. *J Surg Res* **195**, 334-43 (2015).
15. Chai, H., Wang, Q., Huang, L., Xie, T. & Fu, Y. Ginsenoside Rb1 inhibits tumor necrosis factor-alpha-induced vascular cell adhesion molecule-1 expression in human endothelial cells. *Biol Pharm Bull* **31**, 2050-6 (2008).
16. Smolinski, A.T. & Pestka, J.J. Modulation of lipopolysaccharide-induced proinflammatory cytokine production in vitro and in vivo by the herbal constituents apigenin (chamomile), ginsenoside Rb(1) (ginseng) and parthenolide (feverfew). *Food Chem Toxicol* **41**, 1381-90 (2003).
17. Li, X., Chen, J.X. & Sun, J.J. Protective effects of Panax notoginseng saponins on experimental myocardial injury induced by ischemia and reperfusion in rat. *Zhongguo Yao Li Xue Bao* **11**, 26-9 (1990).
18. Li, J. et al. The effects of ginsenoside Rb1 on JNK in oxidative injury in cardiomyocytes. *Arch Pharm Res* **35**, 1259-67 (2012).
19. Liu, D.H. et al. Rb1 protects endothelial cells from hydrogen peroxide-induced cell senescence by modulating redox status. *Biol Pharm Bull* **34**, 1072-7 (2011).
20. Yu, X. et al. Ginsenoside Rb1 ameliorates liver fat accumulation by upregulating perilipin expression in adipose tissue of db/db obese mice. *J Ginseng Res* **39**, 199-205 (2015).
21. Yin, H. et al. Ginsenoside-Rg1 enhances angiogenesis and ameliorates ventricular remodeling in a rat model of myocardial infarction. *J Mol Med (Berl)* **89**, 363-75 (2011).
22. Wang, X.D., Gu, T.X., Shi, E.Y., Lu, C.M. & Wang, C. Effect and mechanism of panaxoside Rg1 on neovascularization in myocardial infarction rats. *Chin J Integr Med* **16**, 162-6 (2010).
23. Dong, G. et al. Rg1 prevents myocardial hypoxia/reoxygenation injury by regulating mitochondrial dynamics imbalance via modulation of glutamate dehydrogenase and mitofusin 2. *Mitochondrion* **26**, 7-18 (2015).
24. Zhu, D. et al. Ginsenoside Rg1 protects rat cardiomyocyte from hypoxia/reoxygenation oxidative injury via antioxidant and intracellular calcium homeostasis. *J Cell Biochem* **108**, 117-24 (2009).
25. Huang, J. et al. Inhibitory Effect of Ginsenoside Rg1 on Vascular Smooth Muscle Cell Proliferation Induced by PDGF-BB Is Involved in Nitric Oxide Formation. *Evid Based Complement Alternat Med* **2012**, 314395 (2012).
26. Gao, Y. et al. Ginsenoside Rg1 inhibits vascular intimal hyperplasia in balloon-injured rat carotid artery by down-regulation of extracellular signal-regulated kinase 2. *J Ethnopharmacol* **138**, 472-8 (2011).

27. Ma, Z.C. et al. Ginsenoside Rg1 inhibits proliferation of vascular smooth muscle cells stimulated by tumor necrosis factor-alpha. *Acta Pharmacol Sin* **27**, 1000-6 (2006).
28. Zhang, H.S. & Wang, S.Q. Ginsenoside Rg1 inhibits tumor necrosis factor-alpha (TNF-alpha)-induced human arterial smooth muscle cells (HASMCs) proliferation. *J Cell Biochem* **98**, 1471-81 (2006).
29. Zhou, Q. et al. Ginsenoside Rg1 inhibits platelet activation and arterial thrombosis. *Thromb Res* **133**, 57-65 (2014).
30. Kimura, Y., Okuda, H. & Arichi, S. Effects of various ginseng saponins on 5-hydroxytryptamine release and aggregation in human platelets. *J Pharm Pharmacol* **40**, 838-43 (1988).
31. Ma, Z.C., Gao, Y., Wang, J., Zhang, X.M. & Wang, S.Q. Proteomic analysis effects of ginsenoside Rg1 on human umbilical vein endothelial cells stimulated by tumor necrosis factor-alpha. *Life Sci* **79**, 175-81 (2006).
32. Song, Y. et al. Ginsenoside Rg1 exerts synergistic anti-inflammatory effects with low doses of glucocorticoids in vitro. *Fitoterapia* **91**, 173-9 (2013).
33. Wang, Y. et al. Ginsenoside Rg1 regulates innate immune responses in macrophages through differentially modulating the NF-kappaB and PI3K/Akt/mTOR pathways. *Int Immunopharmacol* **23**, 77-84 (2014).
34. Du, J., Cheng, B., Zhu, X. & Ling, C. Ginsenoside Rg1, a novel glucocorticoid receptor agonist of plant origin, maintains glucocorticoid efficacy with reduced side effects. *J Immunol* **187**, 942-50 (2011).
35. Lee, H.M., Lee, O.H., Kim, K.J. & Lee, B.Y. Ginsenoside Rg1 promotes glucose uptake through activated AMPK pathway in insulin-resistant muscle cells. *Phytother Res* **26**, 1017-22 (2012).
36. Wang, Y. et al. Ginsenoside Rd attenuates myocardial ischemia/reperfusion injury via Akt/GSK-3beta signaling and inhibition of the mitochondria-dependent apoptotic pathway. *PLoS One* **8**, e70956 (2013).
37. Zeng, X., Li, J. & Li, Z. Ginsenoside Rd mitigates myocardial ischemia-reperfusion injury via Nrf2/HO-1 signaling pathway. *Int J Clin Exp Med* **8**, 14497-504 (2015).
38. Cai, B.X. et al. Ginsenoside-Rd, a new voltage-independent Ca2+ entry blocker, reverses basilar hypertrophic remodeling in stroke-prone renovascular hypertensive rats. *Eur J Pharmacol* **606**, 142-9 (2009).
39. Zhang, Y.X. et al. Ginsenoside-Rd exhibits anti-inflammatory activities through elevation of antioxidant enzyme activities and inhibition of JNK and ERK activation in vivo. *Int Immunopharmacol* **17**, 1094-100 (2013).
40. Wang, L. et al. Inhibitory effect of ginsenoside-Rd on carrageenan-induced inflammation in rats. *Can J Physiol Pharmacol* **90**, 229-36 (2012).
41. Kim, D.H. et al. Ginsenoside Rd inhibits the expressions of iNOS and COX-2 by suppressing NF-kappaB in LPS-stimulated RAW264.7 cells and mouse liver. *J Ginseng Res* **37**, 54-63 (2013).
42. Li, X.F., Shi, X.H. & Luo, Q.Z. Protective effect of ginsenoside Re on myocardial cells of neonatal SD rat subjected to hypoxia injury. *Zhonghua*

*Shao Shang Za Zhi***27**, 169-72 (2011).
43. Xie, J.T. et al. Antioxidant effects of ginsenoside Re in cardiomyocytes. *Eur J Pharmacol***532**, 201-7 (2006).
44. Chen, C.X. & Zhang, H.Y. Protective effect of ginsenoside Re on isoproterenol-induced triggered ventricular arrhythmia in rabbits. *Zhongguo Dang Dai Er Ke Za Zhi***11**, 384-388 (2009).
45. Gao, Y. et al. Inhibitory effects of ginsenoside Re on vascular neointimal hyperplasia induced by balloon-injury and ERK signaling in rats. *Chinese Pharmaceutical Journal***50**, 1589-1593 (2015).
46. Yang, B.R. et al. Pro-angiogenic activity of notoginsenoside R1 in human umbilical vein endothelial cells in vitro and in a chemical-induced blood vessel loss model of zebrafish in vivo. *Chin J Integr Med* (2014).
47. Lee, K.W., Jung, S.Y., Choi, S.M. & Yang, E.J. Effects of ginsenoside Re on LPS-induced inflammatory mediators in BV2 microglial cells. *BMC Complement Altern Med***12**, 196 (2012).
48. Wu, C.F. et al. Differential effects of ginsenosides on NO and TNF-alpha production by LPS-activated N9 microglia. *Int Immunopharmacol***7**, 313-320 (2007).
49. Paul, S., Shin, H.S. & Kang, S.C. Inhibition of inflammations and macrophage activation by ginsenoside-Re isolated from Korean ginseng (Panax ginseng C.A. Meyer). *Food Chem Toxicol***50**, 1354-61 (2012).
50. Lee, I.A., Hyam, S.R., Jang, S.E., Han, M.J. & Kim, D.H. Ginsenoside Re ameliorates inflammation by inhibiting the binding of lipopolysaccharide to TLR4 on macrophages. *J Agric Food Chem***60**, 9595-602 (2012).
51. Zhou, X.M., Cao, Y.L. & Dou, D.Q. Protective effect of ginsenoside-Re against cerebral ischemia/reperfusion damage in rats. *Biol Pharm Bull***29**, 2502-5 (2006).
52. Chen, L.M., Zhou, X.M., Cao, Y.L. & Hu, W.X. Neuroprotection of ginsenoside Re in cerebral ischemia-reperfusion injury in rats. *J Asian Nat Prod Res***10**, 439-45 (2008).
53. Cho, W.C. et al. Ginsenoside Re of Panax ginseng possesses significant antioxidant and antihyperlipidemic efficacies in streptozotocin-induced diabetic rats. *Eur J Pharmacol***550**, 173-9 (2006).
54. Zhang, Z. et al. Ginsenoside Re reduces insulin resistance through inhibition of c-Jun NH2-terminal kinase and nuclear factor-kappaB. *Mol Endocrinol***22**, 186-95 (2008).
55. Han, D.H. et al. Ginsenoside Re rapidly reverses insulin resistance in muscles of high-fat diet fed rats. *Metabolism***61**, 1615-21 (2012).
56. Quan, H.Y. et al. Ginsenoside Re lowers blood glucose and lipid levels via activation of AMP-activated protein kinase in HepG2 cells and high-fat diet fed mice. *Int J Mol Med***29**, 73-80 (2012).
57. He, K. et al. ROCK-dependent ATP5D modulation contributes to the protection of notoginsenoside NR1 against ischemia-reperfusion-induced myocardial injury. *Am J Physiol Heart Circ Physiol***307**, H1764-76 (2014).

58. Zhong, L. et al. Estrogen receptor alpha mediates the effects of notoginsenoside R1 on endotoxin-induced inflammatory and apoptotic responses in H9c2 cardiomyocytes. *Mol Med Rep* **12**, 119-26 (2015).
59. Zhang, W.J., Wojta, J. & Binder, B.R. Notoginsenoside R1 counteracts endotoxin-induced activation of endothelial cells in vitro and endotoxin-induced lethality in mice in vivo. *Arterioscler Thromb Vasc Biol* **17**, 465-74 (1997).
60. Zhang, H.S. & Wang, S.Q. Notoginsenoside R1 from Panax notoginseng inhibits TNF-alpha-induced PAI-1 production in human aortic smooth muscle cells. *Vascul Pharmacol* **44**, 224-30 (2006).
61. Zhang, H.S. & Wang, S.Q. Notoginsenoside R1 inhibits TNF-alpha-induced fibronectin production in smooth muscle cells via the ROS/ERK pathway. *Free Radic Biol Med* **40**, 1664-74 (2006).
62. Yang, Y. et al. Notoginsenoside R1 reduces blood pressure in spontaneously hypertensive rats through a long non-coding RNA AK094457. *Int J Clin Exp Pathol* **8**, 2700-9 (2015).
63. Sun, B., Xiao, J., Sun, X.B. & Wu, Y. Notoginsenoside R1 attenuates cardiac dysfunction in endotoxemic mice: an insight into oestrogen receptor activation and PI3K/Akt signalling. *Br J Pharmacol* **168**, 1758-70 (2013).
64. Jia, C. et al. Notoginsenoside R1 attenuates atherosclerotic lesions in ApoE deficient mouse model. *PLoS One* **9**, e99849 (2014).
65. Ahn, S. et al. Anti-inflammatory activity of ginsenosides in LPS-stimulated RAW 264.7 cells. *Science Bulletin* **60**, 773-784 (2015).
66. Siraj, F.M., SathishKumar, N., Kim, Y.J., Kim, S.Y. & Yang, D.C. Ginsenoside F2 possesses anti-obesity activity via binding with PPARgamma and inhibiting adipocyte differentiation in the 3T3-L1 cell line. *J Enzyme Inhib Med Chem* **30**, 9-14 (2015).
67. Kim, D.H. et al. The inhibitory effect of ginseng saponins on the stress-induced plasma interleukin-6 level in mice. *Neurosci Lett* **353**, 13-6 (2003).
68. Huang, Q. et al. Ginsenoside-Rb2 displays anti-osteoporosis effects through reducing oxidative damage and bone-resorbing cytokines during osteogenesis. *Bone* **66**, 306-14 (2014).
69. Lee, K.T. et al. The antidiabetic effect of ginsenoside Rb2 via activation of AMPK. *Arch Pharm Res* **34**, 1201-8 (2011).
70. Yokozawa, T., Fujitsuka, N., Yasui, T. & Oura, H. Effects of ginsenoside-Rb2 on adenine nucleotide content of rat hepatic tissue. *J Pharm Pharmacol* **43**, 290-291 (1991).
71. Kim, E.J. et al. The ginsenoside-Rb2 lowers cholesterol and triacylglycerol levels in 3T3-L1 adipocytes cultured under high cholesterol or fatty acids conditions. *BMB Rep* **42**, 194-199 (2009).
72. Cho, Y.L. et al. Specific activation of insulin-like growth factor-1 receptor by ginsenoside Rg5 promotes angiogenesis and vasorelaxation. *J Biol Chem* **290**, 467-477 (2015).

73. Chu, S. et al. Ginsenoside Rg5 improves cognitive dysfunction and beta-amyloid deposition in STZ-induced memory impaired rats via attenuating neuroinflammatory responses. *Int Immunopharmacol* **19**, 317-26 (2014).
74. Kim, T.W., Joh, E.H., Kim, B. & Kim, D.H. Ginsenoside Rg5 ameliorates lung inflammation in mice by inhibiting the binding of LPS to toll-like receptor-4 on macrophages. *Int Immunopharmacol* **12**, 110-6 (2012).
75. Lee, S.M. Anti-inflammatory effects of ginsenosides Rg5 , Rz1 , and Rk1 : inhibition of TNF-alpha-induced NF-kappaB, COX-2, and iNOS transcriptional expression. *Phytother Res* **28**, 1893-6 (2014).
76. Lee, Y.Y., Park, J.S., Jung, J.S., Kim, D.H. & Kim, H.S. Anti-inflammatory effect of ginsenoside Rg5 in lipopolysaccharide-stimulated BV2 microglial cells. *Int J Mol Sci* **14**, 9820-33 (2013).
77. Kang, K.S., Kim, H.Y., Yamabe, N. & Yokozawa, T. Stereospecificity in hydroxyl radical scavenging activities of four ginsenosides produced by heat processing. *Bioorganic and Medicinal Chemistry Letters* **16**, 5028-5031 (2006).
78. Tian, J.M. et al. Effect of ginsenoside Rg2 on chemical myocardial ischemia in rats. *Zhongguo Zhong Yao Za Zhi* **28**, 1191-1192 (2003).
79. Cho, Y.S., Kim, C.H., Ha, T.S., Lee, S.J. & Ahn, H.Y. Ginsenoside rg2 inhibits lipopolysaccharide-induced adhesion molecule expression in human umbilical vein endothelial cell. *Korean J Physiol Pharmacol* **17**, 133-7 (2013).
80. Gai, Y., Ma, Z., Yu, X., Qu, S. & Sui, D. Effect of ginsenoside Rh1 on myocardial injury and heart function in isoproterenol-induced cardiotoxicity in rats. *Toxicol Mech Methods* **22**, 584-591 (2012).
81. Lee, E.S. et al. Ginsenoside metabolite compound K differentially antagonizing tumor necrosis factor-alpha-induced monocyte-endothelial trafficking. *Chem Biol Interact* **194**, 13-22 (2011).
82. Jung, J.S., Kim, D.H. & Kim, H.S. Ginsenoside Rh1 suppresses inducible nitric oxide synthase gene expression in IFN-gamma-stimulated microglia via modulation of JAK/STAT and ERK signaling pathways. *Biochem Biophys Res Commun* **397**, 323-328 (2010).
83. Jung, J.S. et al. Anti-inflammatory mechanism of ginsenoside Rh1 in lipopolysaccharide-stimulated microglia: critical role of the protein kinase A pathway and hemeoxygenase-1 expression. *J Neurochem* **115**, 1668-1680 (2010).
84. Gu, W., Kim, K.A. & Kim, D.H. Ginsenoside Rh1 ameliorates high fat diet-induced obesity in mice by inhibiting adipocyte differentiation. *Biol Pharm Bull* **36**, 102-107 (2013).
85. Le Tran, Q. et al. Triterpene saponins from Vietnamese ginseng (Panax vietnamensis) and their hepatocytoprotective activity. *Journal of Natural Products* **64**, 456-461 (2001).
86. Maeng, Y.S. et al. Rk1, a ginsenoside, is a new blocker of vascular leakage acting through actin structure remodeling. *PLoS One* **8**, e68659 (2013).
87. Sun, J. et al. Ginsenoside RK3 Prevents Hypoxia-Reoxygenation Induced

Apoptosis in H9c2 Cardiomyocytes via AKT and MAPK Pathway. *Evid Based Complement Alternat Med***2013**, 690190 (2013).
88. Cho, K., Song, S.B., Tung, N.H., Kim, K.E. & Kim, Y.H. Inhibition of tnf-α-mediated nf-kb transcriptional activity by dammarane-type ginsenosides from steamed flower buds of panax ginseng in hepg2 and sk-hep1 cells. *Biomolecules and Therapeutics***22**, 55-61 (2014).
89. Meng, X. et al. Attenuation of Abeta25-35-induced parallel autophagic and apoptotic cell death by gypenoside XVII through the estrogen receptor-dependent activation of Nrf2/ARE pathways. *Toxicol Appl Pharmacol***279**, 63-75 (2014).
90. Meng, X.B. et al. P90RSK and Nrf2 Activation via MEK1/2-ERK1/2 Pathways Mediated by Notoginsenoside R2 to Prevent 6-Hydroxydopamine-Induced Apoptotic Death in SH-SY5Y Cells. *Evid Based Complement Alternat Med***2013**, 971712 (2013).
